# Male Urinary Incontinence Severity and Non-Adjustable Sling Outcomes: Protocol for a Multivariate Dose–Response Meta-Analysis

**DOI:** 10.3390/jcm15114140

**Published:** 2026-05-27

**Authors:** Emilio Sacco, Francesco Pio Bizzarri, Marco Campetella, Lorenzo D’Amico, Filippo Gavi, Pierluigi Russo, Riccardo Bientinesi, Maria Chiara Sighinolfi, Bernardo Cesare Maria Rocco

**Affiliations:** 1 Department of Urology, Ospedale Isola Tiberina-Gemelli Isola, Università Cattolica del Sacro Cuore, 00168 Rome, Italy; 2 Department of Urology, Fondazione Policlinico Universitario Agostino Gemelli IRCCS, Università Cattolica del Sacro Cuore, 00168 Rome, Italy; 3 Department of Urology, Mater Olbia Hospital, 07026 Olbia, Italy

**Keywords:** dose–response, male, meta-analysis, prostatectomy, protocol, splines, systematic review, urinary incontinence, urethral sling

## Abstract

**Background and Objective:** Male stress urinary incontinence (UI), particularly after radical prostatectomy, remains a clinically relevant condition with a detrimental impact on patients’ quality of life. Non-adjustable male slings are widely used for the treatment of this condition. Although baseline UI severity is an established predictor of sling outcomes, the true relationship between UI severity and sling efficacy remains unknown. This protocol describes a systematic review with dose–response meta-analysis designed to investigate the association between UI severity and the outcomes of non-adjustable slings. **Methods:** A comprehensive literature search will be conducted in MEDLINE (PubMed), Web of Science, Scopus, and the Cochrane Central Register of Controlled Trials from database inception. Eligible studies will include randomized controlled trials and observational studies enrolling adult men undergoing sling surgery, with at least six months of follow-up. UI severity must be measured preoperatively using objective metrics and reported across at least two severity categories. The primary outcomes will be failure to achieve cure or failure to achieve overall success defined as cure or clinically meaningful improvement. Study quality will be assessed using the QUIPS tool. Dose–response relationships will be analyzed using advanced meta-analytic methods, allowing assessment of both linear and nonlinear associations. Potential sources of heterogeneity will be explored through subgroup analyses and meta-regression. The robustness of the findings will be evaluated through sensitivity analyses and assessment of publication bias. Finally, the certainty of evidence will be graded using the GRADE framework. We will adhere to the PRISMA recommendations in the reporting of this review. **Conclusions:** This analysis will provide quantitative evidence to improve patient selection, refine counseling, and support evidence-based decision-making in the surgical management of male stress UI.

## 1. Introduction

Male stress urinary incontinence (UI) is associated with a substantial impact on quality of life, with post-prostatectomy UI being the most common etiology, even in the era of advanced robotic surgery [1,2,3,4]. Following radical prostatectomy for prostate cancer, UI can affect up to 46% of patients, with rates varying according to surgical technique, surgeon experience, time since surgery, and the definition of UI [5,6]. While most patients experience spontaneous improvement within the first 12 months post-operatively, persistent UI beyond this period significantly impacts physical, psychological, and social well-being [7,8].

Post-prostatectomy UI is a complex condition primarily related to sphincter deficiency [9,10]. Conservative management, including pelvic floor muscle training, biofeedback, and pharmacological therapies, should be attempted as first-line therapy [11,12,13]. However, when conservative measures fail, surgical intervention becomes necessary. The surgical options include male slings and artificial urinary sphincters (AUS). AUS is traditionally considered the gold standard for severe UI [14,15,16,17].

Male synthetic slings have gained increasing popularity as a less invasive alternative to AUS, particularly for mild-to-moderate UI. Several sling types are available, including non-adjustable (fixed) and adjustable slings, with non-adjustable slings being the most frequently implanted [1,18,19]. The reported success rates range widely from 33% to 90% [18,19,20,21].

Given the variability in outcomes, identifying predictors of efficacy remains clinically important. The severity of UI varies widely among patients, ranging from minimal stress-related leakage during vigorous activity to severe continuous leakage requiring multiple absorbent pads daily. Multiple studies and a meta-analysis have identified baseline UI severity as a critical determinant of sling outcome, with higher preoperative pad usage and daily urinary leakage volume associated with increased failure rates [20,22,23]. In addition to baseline UI severity, other reported risk factors include prior radiation therapy, previous UI treatments, history of urethral strictures, and detrusor overactivity [20].

Despite extensive literature on male slings, the relationship between UI severity and treatment success remains incompletely characterized. Most studies classify patients into two or three arbitrary severity categories, potentially obscuring important dose–response patterns. Even within the “moderate” severity category, there exists substantial heterogeneity in outcomes, suggesting that more granular severity stratification could improve prognostication [24]. These limitations highlight the need for a comprehensive re-evaluation of the dose–response relationship between UI severity and sling outcomes.

### Rationale and Objective

Current clinical decision-making regarding male sling candidacy relies heavily on categorical severity classifications (mild, moderate, severe), often using arbitrary cutoffs for pad count or leakage volume [25]. While clinically practical, this approach may result in substantial loss of information, as continuous variability in baseline severity is reduced to discrete groups. This approach may also introduce misclassification, particularly for patients near category boundaries, and may bias effect estimates by assuming homogeneity within categories. Furthermore, traditional meta-analyses based on categorical comparisons are unable to capture nonlinear relationships or identify threshold effects. These limitations may partially explain the heterogeneity and inconsistency of reported results. While the FORESEE meta-analysis successfully identified UI severity as a key prognostic factor, it failed to investigate the shape of the relationship between UI severity and risk of failure and could not definitively establish whether treatment effectiveness declines gradually or demonstrates sharp threshold effects [20]. A dose–response meta-analysis overcomes these limitations, because it offers several advantages over conventional approaches. First, it synthesizes data across the entire range of exposure rather than relying on predefined categories, allowing for a more precise and clinically informative assessment of the relationship. Second, it can detect and characterize nonlinear relationships that may reveal clinically meaningful thresholds for treatment success. Third, it maximizes statistical efficiency by incorporating all available data points rather than collapsing continuous measures into categories [26,27]. By systematically combining relevant literature, this review aims to perform a dose–response analysis examining the relationship between UI severity and male non-adjustable sling outcome. By incorporating all available evidence into clinical practice, our results will assist healthcare professionals in patient selection and counseling, substantially informing treatment decision-making.

## 2. Materials and Methods

### 2.1. Research Question

The review question was defined according to the PICOTS system as proposed by the CHARMS checklist and subsequent improvement (Table 1) [28].

### 2.2. Registration and Reporting

The protocol will be registered in PROSPERO prior to the initiation of study selection, eligibility assessment, and data extraction, ensuring full prospective registration in accordance with best practice recommendations. We followed the Preferred Reporting Items for Systematic Reviews and Meta-Analyses (PRISMA) recommendations for the reporting of this systematic review protocol (see Supplementary Table S1) [29], and will adhere to the PRISMA principles as modified by Xu et al. specifically for dose–response meta-analyses during the reporting of the review [30,31]. In performing this review, we will also follow the Cochrane handbook for systematic reviews of interventions [32], the recommendations of the Cochrane Prognosis Methods Group [33,34,35], and other guidance for specifically conducting systematic reviews and meta-analyses of prognostic studies [36].

### 2.3. Literature Search Strategy

A systematic literature search will be conducted searching the following electronic databases from inception: MEDLINE (via PubMed), Web of Science, Scopus, and Cochrane Central Register of Controlled Trials (CENTRAL). The searches will not be limited by historical time constraints. The search terms and strategies are reported in Supplementary Tables S2–S5.

The key strategies to optimize signal-to-noise ratio will be:

1. Sensitivity versus precision: the search strategy will combine controlled vocabulary terms (MeSH terms for PubMed) and free-text keywords related to:
  - Male UI and post-prostatectomy UI context;
  - Male slings, suburethral slings, retrourethral sling, transobutator sling, bone-achored sling;
  - Specific device names.

These strategies prioritize sensitivity to capture all relevant studies, with manual screening improves precision.

2. Intervention-focused approach:
  - Primary focus on sling-specific terms;
  - Emphasis on device names and sling types most likely to appear in relevant studies.
3. Enhanced exclusions:
  - Comprehensive female sling and female-specific procedure exclusion terms;
  - Exclude non-relevant prostatectomy complication papers;
  - Strict publication type filtering.
4. Retained sensitivity:
  - To capture variations in terminology (with/without hyphens, spacing variations);
  - Includes both brand names and generic sling descriptions;
  - Minimal language and no date restrictions.
5. Updates: searches will be re-run before final submission to capture newly published studies.
6. Publication bias minimization:
  - Citation tracking: Citation tracking will include backward reference list screening of all included studies and relevant reviews, as well as forward citation searching using databases such as PubMed and Web of Science. The ‘similar articles’ function in PubMed will also be used to identify additional relevant studies.
  - Gray literature search: To minimize publication bias, additional searching will include ClinicalTrials.gov and the WHO International Clinical Trials Registry Platform using predefined search terms (‘incontinence’ AND ‘male sling’). Conference abstracts from major urological meetings (EAU, AUA, ICS, SUFU) from the last 5 years will be screened. Manufacturer websites (Boston Scientific, Coloplast, APIS, pfmmedical, Aspide Medical) will be searched for relevant study reports.

### 2.4. Eligibility Criteria

Applicability to the review question will be based on the following criteria:

- Inclusion criteria:
- Use of synthetic male slings.
- Any study design (randomized controlled trials, prospective and retrospective cohort studies, case series).
- Minimum sample size of 30 patients.
- Adult male patients (age ≥ 18 years).
- Minimum follow-up of 6 months.
- The index prognostic factor (baseline UI severity) must be:
  1. Measured before sling implantation.
  2. Assessed using objective measures.
  3. Stratified by at least two categories.
  4. Clearly defined with category boundaries in a way that allows dose assignment.
- Studies must report both the number of patients and number of cases (failures) for each UI category to avoid biases in the estimates for the variances.
- Explicit definition of treatment failure/success criteria.
- For studies published by the same research group, the study will be selected according to the following criteria, in order of priority: (1) the largest one; (2) the study with the most adjusted effect measures; (3) the study with the longest follow-up. Multiple studies by the same research group may still be included if reported UI severity measure–outcome combinations are different between papers (e.g., data on ‘cure’ reported in a paper and data on ‘cure plus improvement’ reported in another).
- Conference abstracts will be considered for inclusion if they provide sufficient methodological detail and extractable outcome data, or if additional data can be obtained from the authors.
- Language restrictions will be minimized by accepting English, Spanish, French, and Italian articles, with non-English articles translated as necessary.

### 2.5. Exclusion Criteria

- Use of adjustable slings (due to fundamentally different mechanisms of action of these devices).
- Use of absorbable or biodegradable sling materials.
- Follow-up duration less than 6 months.
- Less than 30 analyzed patients.
- Analysis of re-do procedures.
- Mixed populations where male sling data (e.g., adjustable and non-adjustable slings) cannot be separated.
- Studies reporting insufficient data and whose additional data will not be obtained on request.
- Incomplete or unclear UI severity categorization and studies providing an effect size analyzed as a continuous variable only (these studies will be used for sensitivity analysis if the linear trend is selected as the best final dose–response model).
- The citation is a case report, a review or meta-analysis, or a book chapter or thesis.
- The citation that is an expert consensus, editorial, letter, comment, animal study, communication, or abstract lacking sufficient data for analysis will be excluded.
- Duplicated studies not fulfilling the inclusion criteria for studies published by the same research group.

### 2.6. Study Selection

A two-stage selection process will be undertaken. As a first step, two independent reviewers will screen titles and abstracts using predefined eligibility criteria. At the second step, full-text articles of potentially eligible studies will be retrieved and assessed independently by two reviewers that will independently perform the selection process without consideration for the results. Both screening stages will be piloted on a random selection of 10 citations. During both stages, disagreements will be resolved through discussion among reviewers and, if necessary, with consultation with a third reviewer until mutual agreement is reached. All citations will be recoded in Zotero 5.0. A PRISMA flow diagram will document the selection process, including reasons for exclusion at the full-text review stage.

### 2.7. Data Extraction

Key information from included studies will be extracted according to the CHARMS checklist [28] in its modified version suitable for reviews of prognostic factors (CHARMS-PF) [37]. Data will be extracted independently by two reviewers using a standardized, pilot-tested data extraction form to ensure consistency, and recorded in a Microsoft Excel spreadsheet. The spreadsheet will be created by two reviewers and thereafter pilot-tested and finalized on a random selection of five studies. The form will be reviewed and modified, if necessary, after group discussion.

### 2.8. Data Items

The major groups of variables to be coded for each eligible study are as follows: (1) study’s general information (e.g., first author, journal, year of publication, country, study frame and design, sample size, follow-up duration, funding source and conflicts of interest), (2) participant characteristics (e.g., inclusion/exclusion criteria, number of eligible and treated patients, age at baseline, UI etiology and categorization, number of patients and failures within each UI severity category, other risk factors), (3) intervention characteristics (e.g., type of sling, any comparator treatment, surgical technique), (4) outcomes (e.g., outcomes types and definitions, endpoints, event rates, attrition) and (5) other relevant quantities (measurement methods, type of statistical analyses such as univariable versus multivariable, set of variables in the multivariable model, modeling method, effect size estimates with variance and *p*-value).

### 2.9. Risk of Bias Assessment

The methodological quality and risk of bias (RoB) of included studies will be assessed using an adapted version of the Quality in Prognostic Studies (QUIPS) tool [37]. This tool has demonstrated satisfactory reliability and evaluates six domains: (1) study participation, (2) study attrition, (3) prognostic factor measurement, (4) outcome measurement, (5) study confounding, and (6) statistical analysis and reporting. To enhance consistency and reproducibility, the QUIPS domains and their prompting items were predefined and operationalized a priori for this review, to adapt the prompting items for our specific review question according to recommendations on RoB assessment (Supplementary Materials) [35,37,38]. Each domain will be rated as low, moderate, or high RoB.

This classification is based on the criteria proposed by Grooten et al. [38]: a study satisfying low RoB in at least five domains and without any high-risk rating will be designated as a study with an overall low RoB. If one or more domains are classified as high RoB, or ≥3 moderate RoB, then this paper will be classified as high RoB. All papers in between will be classified as having moderate RoB (Supplementary Table S6).

Two authors will independently perform the quality assessment after a piloted screening of five studies. They then will meet and review their judgments for agreement. If agreement cannot be reached, a third author will render the decision. Final judgments will be based on a structured qualitative assessment of the predefined items, incorporating reviewer expertise and the overall methodological context of each study. Overall and study-level RoB assessment will be displayed using the ROBVIS V2 web app [39].

### 2.10. Exposures and Comparators

Exposures will include any preoperative quantitative UI metrics evaluated for association with sling failure. Based on previous studies [20], 24 h pad count and 24 h leakage volume (measure by pad weighing) are expected to be the most frequently reported UI metrics. Comparators will be higher levels of UI severity. Given the lack of validated methods to directly convert between different severity metrics, separate dose–response analyses will be conducted for each exposure measure (e.g., pad count, pad test leakage volume, grading scales) and no pooled curve across non-equivalent exposure measures will be generated. Within each analysis, exposure levels will be harmonized using standard dose assignment methods, ensuring comparability across studies using the same metric.

### 2.11. Outcome Measures

The primary outcomes will be treatment failure to achieve cure (FoC) or failure to achieve overall success (FoS). Cure will include complete (“dryness”) or nearly complete continence, while overall success will include cure or at least a clinically meaningful improvement of the baseline UI status (often called “social continence”). Methods of measurement will be as per included studies. Heterogeneity in definitions of outcomes is expected in sling literature [20]. Therefore, our approach will aim to minimize this variability by selecting the most comparable definitions or, when feasible, recalculating outcomes using consistent criteria according to a predefined hierarchical framework based on objective measures and commonly used patient-reported outcomes [20]:

- FoC:
  1. Primary definitions:
    - Persistence of any degree of UI (not dry).
    - >2 g of leakage volume at 24 h pad test.
    - >1 g at 1 h pad test.
    - Use of any pad.
  2. Secondary acceptable definitions:
    - Use of any pad except for safety pad.
    - Less than ‘very much improved’ rating at Patient-Global Impression of Improvement (PGI-I) questionnaire (score < 5).
    - Positive stress test.
    - >5 g of leakage volume at 24 h pad test.
    - <90% improvement of 24 h leakage volume.
    - Any self-reported UI at validated UI score/questionnaire.
    - Any other study-specific failure criteria judged close to above-mentioned definitions.
- FoS:
  1. Primary definitions:
    - <50% improvement in pad weight.
    - <50% improvement in pad count.
  2. Secondary acceptable definitions:
    - Persistent use of >1 pad per day.
    - >25 g of leakage volume at 1 h pad test.
    - >50 g of leakage volume at 24 h pad test.
    - Less than ‘much’ or ‘very much’ improved rating at PGI-I (score < 4).
    - No decrease in pads use.
    - No decrease in leakage volume.
    - No patient satisfaction.
    - Any other study-specific failure criteria judged close to above-mentioned definitions.

When multiple leakage volume-based definitions are available, priority will be given to 24 h pad test measurements, as they better reflect daily urinary leakage.

For each included study, the selected outcome definition and follow-up time point will be explicitly reported in a study-level table; any study-specific outcome definitions outside the predefined hierarchy will be conservatively applied, included only if clearly comparable, and documented with explicit justification.

### 2.12. Effect Measure

Dose-level odds ratio (OR) with 95% confidence intervals (CI) comparing failure rates across baseline UI severity categories will be extracted as reported in the studies. When reported, OR for linear trend will also be extracted.

Although most reported effect measures are expected to be derived from univariable (unadjusted) analyses, we will primarily focus on adjusted estimates, as they better reflect the independent contribution of risk factors [34,35]. We will perform separate meta-analyses based on adjusted (‘adjusted meta-analysis’) and unadjusted (‘unadjusted meta-analysis’) effect measures for both continence outcomes. Studies will be included in the adjusted meta-analysis only if adequate statistical control is provided to account for the effect of relevant covariates on the association between UI severity and outcomes. When studies report multiple outcome time points, the longest available follow-up will be used as the primary endpoint. If different multivariable models are reported, we will select the maximally adjusted model to reduce the risk of possible unmeasured confounding. For sensitivity analyses, outcomes at different follow-up endpoints will be extracted separately when available. Adjustment for prior pelvic irradiation will be considered mandatory for defining adequately adjusted models during study quality assessment, because it is a major prognostic factor of sling efficacy with high certainty in evidence [20]. Other clinically relevant factors such as prior urethral interventions or incontinence surgery will not be required due to the currently limited and moderate-certainty evidence supporting their independent prognostic role [20].

**Figure 1:**
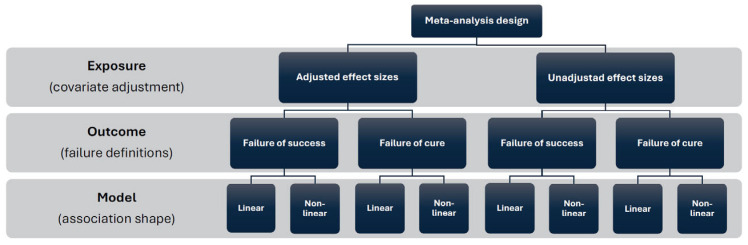
Meta-analysis design.

The meta-analysis design is schematically displayed in Figure 1.

When not directly reported, unadjusted OR with 95% confidence intervals (CI) will be calculated from the raw data (number of events and non-events in each severity category). The lowest severity category will be used as the reference group. When zero counts occur in a cell of a 2 × 2 contingency table to calculate the OR, the Haldane–Anscombe correction will be applied [40]. When not directly reported, standard error will be calculated using CI or *p*-value, according to Altman and Bland [41]. For studies reporting a category other than the lowest one as a reference, we will recalculate the OR assuming the lowest category as the reference. OR with 95% CI will also be used as the synthesized measure of effect size. The association will be reported following the convention that OR > 1 indicates that UI severity is associated with an increased risk of failure, and OR < 1 indicates that UI severity has a protective effect.

### 2.13. Dose Assignment

For each baseline severity category, a representative dose will be assigned using the following hierarchical approach [42,43,44]:

1. Parametric method: when studies report mean or median values within categories, these will be used directly.
2. Non-parametric method: when only category boundaries are provided, the midpoint of the category will be calculated as the representative dose.

For open-ended categories, we will use the following approach:

- For open-ended smallest category, the median (or the midpoint) is set as the dose assuming that the beginning is zero (e.g., 5 if <10);
- For open-ended largest category, the median (or the midpoint) of the previous category minus the starting value of the previous category is added to the starting value of the last category, and the ensuing value is set as the dose.

### 2.14. Statistical Analysis

#### One-Stage Dose–Response Approach

We will employ the one-stage dose–response multivariate meta-analysis framework implemented in dosresmeta package for R software 4.6, as described by Crippa and Orsini [45]. This approach offers several advantages over traditional two-stage methods [46,47,48]. In particular, it allows for the inclusion of studies reporting only two categories, while the traditional two-stage method typically necessitates a minimum of three levels for the independent variable, thus increasing the number of included studies.

The one-stage model will be implemented using generalized least squares regression with a random-effects structure to account for both within-study and between-study variability. The model fitting will use the restricted maximum likelihood (REML) random-effects estimator.

### 2.15. Linear and Nonlinear Models

Both linear and nonlinear dose–response models will be fitted. We will first assume a linear response model for the association between UI severity and sling outcomes. The nonlinear model will employ restricted cubic splines with three knots positioned at fixed percentiles of the dose distribution. Restricted cubic splines are well suited for modeling complex curvilinear associations [49]. A restricted cubic splines model with three knots balances flexibility and adequate data coverage across the dose range against overfitting risk in meta-analysis contexts where the number of studies may be limited [50]. This model is defined by two coefficients [51]. It is standard practice to position the three knots at the 10th, 50th, and 90th percentiles of the independent variable to ensure balanced coverage of the exposure range [50,52,53].

Following methodological recommendations for dose–response meta-analysis, we will fit linear models if at least three studies are available, though 6–8 studies are preferred for more stable estimates [45,53]. Nonlinear models using restricted cubic splines will only be fitted if at least 8–10 studies (according to their quality and the number of exposure categories) are available to ensure model stability and reliable parameter estimation [45,54]. If these criteria are not met, analyses will be restricted to linear models or summarized narratively (see ‘Section 2.25’).

For nonlinear models, the “overall *p*-value”, derived from testing the null hypothesis that both regression coefficients are equal to zero, will assess the overall significance of the dose–response relationship. Departure from linearity will be assessed by testing the null hypothesis that the coefficient of the second spline is equal to zero, yielding the “nonlinearity *p*-value” [51]. A multivariate extension of univariate Wald-type test will be used for hypothesis testing [45].

### 2.16. Heterogeneity Assessment

Inherent between-study clinical and methodological heterogeneity is expected in meta-analyses of observational studies [20]. Clinical heterogeneity is assumed to be mainly derived from the different types of treatment (e.g., different sling types and surgical techniques) and different patient characteristics (e.g., age, UI etiology, and severity) among included studies, while methodological heterogeneity may be due to a variety of study designs (e.g., prospective and retrospective) and diverse follow-up lengths and sample sizes.

Between-study heterogeneity will be quantified using the Variance Partition Coefficient (VPC), calculated as:

VPC = τ^2^/(τ^2^ + σ^2^)

where τ^2^ represents between-study variance and σ^2^ represents average within-study variance [27,53]. The VPC has advantages in dose–response meta-analysis compared to traditional I^2^ statistics. The VPC provides an absolute measure of the proportion of total variation attributable to heterogeneity and is more interpretable in mixed-effects, models remaining stable across different dose ranges [54]. The best practice is to plot the VPC against observed dose levels: a flat VPC curve suggests that heterogeneity is constant across all doses, while a rising or falling VPC curve indicates that certain exposure ranges are more prone to study-level differences (e.g., different populations responding differently only at high doses) [27]. The VPC values will be interpreted according to the following thresholds: <0.30 (low heterogeneity), 0.30–0.70 (moderate heterogeneity), and >0.70 (substantial heterogeneity) [46,54,55,56].

We will overlay a locally weighted scatterplot smoother (LOWESS) to examine how the between-studies heterogeneity changes over the exposure range [27]. Subgroup analysis and meta-regression will be used to address the cause of heterogeneity (see below).

### 2.17. Model Selection

The best-fitting model will be selected based on the Akaike Information Criterion (AIC), a measure of relative model quality that balances goodness-of-fit against model complexity, with lower values indicating better models. AIC penalizes additional parameters to prevent overfitting while rewarding improved fit [56]. A difference in AIC of >10 points will be considered strong evidence favoring the lower valued model, while differences of 4–7 points suggest moderate evidence and differences of <2 points indicate minimal difference [57].

For more insightful and comprehensive assessment of whether the pooled dose–response relation adequately summarizes the published results, we will also evaluate:

- Bayesian Information Criterion (BIC). Similar to AIC but applies a stronger penalty for model complexity, making it more conservative in selecting complex models. BIC is particularly useful when the true model is expected to be parsimonious [57].
- Dose-group-level multivariate decorrelated residuals analysis. Visual assessment of model adequacy by plotting dose-group-level decorrelated standardized residuals (provided by the dosresmeta framework) against exposures. Residuals are transformed using the Cholesky factorization of the variance–covariance matrix [58]. The use of transformed (decorrelated) residuals is required because the multi-dose data points within each study are inherently correlated. The interpretation of this scatterplot is analogous to that of the residual-versus-predictor-plot that is used as a goodness-of-fit tool after classic ordinary least squares regression. If the fit is perfect, all the points will lie on the horizontal (zero) line (reference line). Systematic patterns suggest model misspecification, while random scatter around zero across the entire dose range indicates adequate fit [59]. We will overlay a LOWESS line to help discern possible patterns.
- Deviance (D). Measure of the total absolute difference between reported and predicted (log) effect size, considering the covariance structure of the residuals. The smaller the deviation, the closer the reported and fitted log(OR) will be [60].
- Adjusted coefficient of determination (R^2^adj) based on decorrelated data. Standardized measure of the degree of agreement between model predictions and empirical data [61]. It measures the proportion of variability accounted by the dose–response model, and ranges from 0 (no dose–response association) to 1 (perfect fit).

This complementary diagnostic detects potential misspecification (e.g., nonlinearity, heteroscedasticity) or influential observations not captured by relative AIC comparisons, ensuring robust inference.

### 2.18. Metabiases

#### Publication Bias

Potential publication bias and small-study effects will be assessed using a residuals-based analysis combining graphical inspection and statistical testing, adapted for the multivariate nature of one-stage dose–response meta-analysis:

- Graphical inspection. Contour-enhanced funnel plots will be constructed using the decorrelated standardized residuals derived from the goodness-of-fit assessment of the one-stage model [59]. Unlike standard funnel plots that plot the effect size, these plots utilize residuals against their precision (inverse of the standard error). Shaded regions (contours) will indicate the *p* < 0.10, *p* < 0.05, and *p* < 0.01 significance levels. This allows for the differentiation of asymmetry caused by publication bias (missing non-significant studies in the “hollow” regions of the funnel) from other sources of heterogeneity or model misspecification.
- Statistical testing. Statistical evidence of funnel plot asymmetry will be evaluated using Egger’s linear regression test [60]. Standard Egger’s tests violate the assumption of independent observations. In the context of the one-stage approach, the test is better performed by regressing the decorrelated residuals against their precision [58]. By using the decorrelated residuals, the error terms are independent and identically distributed, satisfying the assumptions of the linear regression model. An intercept significantly different from zero will be considered evidence of significant small-study effects. A positive intercept suggests that smaller studies (low precision) tend to report higher residuals than larger studies. A negative intercept suggests that smaller studies tend to report lower residuals than larger studies. Due to the traditionally low power of tests for small-study effects, a significance threshold of *p* < 0.10 will be used to denote significant asymmetry. To distinguish real publication bias from unsatisfactory model fit (misspecification), Egger’s test will be performed using the best fitted one-stage meta-analytic model based on model selection methods.
- Sensitivity analysis for small-study effect. In case of evidence of publication bias using the best fitted model, we will perform one-stage meta-regression including study-level sample size as a moderator. This will quantify the impact of study precision on the pooled dose–response slope. If the coefficient for the sample size is statistically significant, it supports “small-study effects”.

All these analyses will be performed only on meta-analyses featuring 10 or more studies [61].

### 2.19. Selecting Reporting

We will assess the risk of selective reporting within individual studies by comparing the outcomes reported in the methods and results sections of each included study. Where available, we will compare published reports with study protocols or trial registrations (e.g., entries in ClinicalTrials.gov) to identify discrepancies such as omission of prespecified outcomes, addition of non-prespecified outcomes, or incomplete reporting of outcomes.

Selective reporting will be evaluated as part of the overall risk of bias assessment. Any suspected selective reporting will be considered when interpreting the results of the review.

### 2.20. Sensitivity Analyses

To assess the robustness of findings, the following sensitivity analyses will be conducted:

- Using midpoint dose assignment for all studies (rather than median/mean when available).
- Excluding studies with extreme open-ended upper categories of UI (e.g., >8 pads/day, >800 mL/day), as they may disproportionately influence the curve shape.
- Using outcomes at different follow-up endpoints, if reported.
- Restricting the analyses to the most homogeneous outcome definitions.
- Including studies reporting linear trends only (excluded from our one-stage methodology), by using a two-stage dose–response random-effects approach and comparing the result with the primary one-stage approach (for linear trend estimation only) [46,47].
- Setting knots for spline curves at different doses, such as repositioning spline knots at the 25th, 50th, and 75th fixed percentiles and using data-driven knot selection based on the distribution of observed doses. We will examine the quality of the different models.
- Excluding studies in the form of randomized controlled trial or published as abstract only.
- Outliers/influential studies analyses (see below).

### 2.21. Outliers/Influential Studies Analyses

For the assessment of potential outliers and influential data sources that may impact the robustness of the findings, multiple diagnostic measures will be applied when at least three studies will be available to ensure that the conclusions do not hinge on a few unusual studies:

1. Leave-one-out analysis. To assess the stability of our findings and evaluate whether the meta-analysis results are biased by any individual study, we will iteratively remove one study at a time and recalculate the pooled dose–response relationship using the one-stage random-effects approach [62,63]. For linear models, the results will be considered robust if no changes in direction and statistical significance of the overall effect size are observed on the exclusion of any studies, and the estimates in each case are well within the CIs of the overall estimate. For nonlinear models, restricted cubic splines will be re-fitted in each iteration to evaluate the robustness of the curve’s architecture. Influence will be quantified by observing the shift in second model coefficients. To maintain computational stability and comparability across iterations, knots will be fixed at the same percentiles as the primary model. For both linear and curvilinear models, we will also perform a visual inspection of the plot (“spaghetti plot”) overlaying the predicted curve from the full model with the curves of the leave-one-out models. If one curve (representing the exclusion of study X) deviates significantly from the bundle, study X is influential.
2. Dose-group-level multivariate decorrelated residuals analysis. The above-mentioned goodness-of-fit analysis of residuals at the dose-group-level will also assist in detecting specific dose levels within studies that act as outliers [58].
3. Study-level multivariate decorrelated residuals analysis. Identifies outlier studies with outcomes that differ significantly from the predicted value based on the fitted dose–response model [60]. Residuals exceeding a Z-score of +/−1.96 will be considered indicative of a significantly poor fit.
4. Cook’s distance. The influence of individual studies on the pooled estimates will be quantified using Cook’s distance [60], calculated via the leave-one-out procedure. Studies exceeding the threshold of 4/n will be classified as highly influential. The relationship between study-level fit and global influence will be visualized using a diagnostic map, allowing for the identification of outliers that disproportionately affected the overall dose–response relationship.
5. Re-analysis of the dose–response association excluding outliers and influential studies.

### 2.22. Subgroup Analyses and Meta-Regression

If substantial heterogeneity is observed, and at least 10 studies are available, subgroup analyses and multivariate meta-regressions will be conducted to explore the potential sources of heterogeneity [34]. Subgroup analysis will consider the following study-level dichotomous covariates:

- Risk of bias: comparing studies with low-to-moderate versus high risk of bias studies.
- Study design (retrospective vs. prospective).
- Sling type: comparing Advance/AdVance XP slings versus other sling types.
- Outcome definition (primary and more stringent versus secondary definitions).
- Follow-up duration: shorter term (<12 months) versus longer term (≥12 months) follow-up.

Meta-regression will use the following continuous covariates:

- Publication year.
- Sample size.
- Mean follow-up duration.
- Number of confounder adjustments.

Given the potential for overfitting with multiple covariates and limited numbers of studies, each covariate will be examined in separate univariable models, with multivariable models constructed only if at least 10 studies per covariate are available [64].

The statistical significance of differences between subgroups and the influence of continuous covariates will be evaluated using a multivariate Wald-type test for moderators, with *p* < 0.10 considered statistically significant given the reduced power of such tests.

We will also evaluate if the statistically significant moderators improve the model quality and significantly reduce the VPC explaining the observed heterogeneity.

### 2.23. Certainty of Evidence

The certainty of evidence will be assessed using the Grading of Recommendations Assessment, Development, and Evaluation (GRADE) framework, adapted for prognostic factor research [64,65].

Evidence will be rated as high, moderate, low, or very low certainty across the dose–response curves for clinically meaningful exposure contrasts across the severity spectrum and, where appropriate, for different ranges of UI severity. The certainty assessment will focus on the presence, magnitude, and consistency of the dose–response relationship rather than solely on statistical significance.

The assessment of a body of observational studies in the field of prognostic research begins as high certainty in the evidence [65,66]. The GRADE approach considers five factors that can decrease the confidence in estimates of effects: RoB, inconsistency (VPC > 70%), indirectness, imprecision, and publication bias. If no serious limitations are present in any of these areas, three considerations may warrant upgrading:

- Dose–response gradient. The presence of a clear dose–response relationship may increase certainty by one level, as it strengthens causal inference.
- Large effect size. An upgrade for a large magnitude of effect will be considered if the predicted relative risk (RR) reaches a threshold of >2.0 when comparing a high-exposure level (e.g., the 90th percentile of observed dose) to the low-exposure level (e.g., 10th percentile of observed dose). This threshold reflects effects of sufficient magnitude that residual confounding is unlikely to entirely explain the observed association. To ensure the upgrade is justified, we will also require that the 95% CIs at these dose levels demonstrate adequate precision and that the effect is consistent across the primary studies without evidence of serious residual confounding.
- Plausible confounding that would increase confidence in an estimate [66,67].

We will downgrade for RoB if more than 50% of the studies are at high RoB or if the results are no longer significant or even reversed after removing high RoB studies. Imprecision will be evaluated by examining the width of CI around predicted effect estimates at clinically relevant severity levels. If the meta-analysis shows a significant dose–response gradient and a significant effect, we will only upgrade once unless the evidence is exceptionally strong (e.g., RR > 5.0). Meta-analysis unstable at sensitivity analysis or results based on a single study will start as “moderate” certainty.

Grade assessment will be conducted in duplicate with disagreements resolved through discussion and, if necessary, with the senior reviewer (ES) until mutual agreement is reached.

### 2.24. Missing Data

For missing data, study investigators will be contacted for unreported key data or additional information via electronic mail and/or researchgate.net, if possible. Missing data distribution is a common feature of meta-analyses of continuous data. Calculation of missing mean values and standard deviation will be based on Wan’s methods [68]. Data displayed in graphs will be extracted when not retrieved otherwise using WebPlotDigitizer software V4 (URL https://automeris.io/WebPlotDigitizer/ accessed on 18 January 2025). If necessary, imputation of the average value borrowed from one or more studies in the meta-analysis will be used for standard deviation [68,69].

### 2.25. Contingency Plan for Sparse Data

Given the expected heterogeneity and potential fragmentation of the literature, a prespecified hierarchical strategy will be applied to address sparse data scenarios. Analyses will be conducted according to data availability, prioritizing robustness and interpretability. Dose–response models will be fitted only when enough studies with comparable exposures and outcomes are available. Linear models will be preferred in smaller datasets, while nonlinear models will be restricted to adequately powered scenarios. Subgroup analyses, meta-regression, and publication bias assessments will be performed only when supported by sufficient data. If data become sparse after stratification, analyses will be simplified using linear approaches or descriptive synthesis, rather than fitting unstable models.

### 2.26. Software and Statistical Packages

All analyses will be conducted using R software (RStudio v.2026.01.1+403). The dosresmeta (version 2.2.0) package will be used for one-stage dose–response meta-analysis [70,71,72].

Additional packages will include mvmeta (version 1.0.3), meta (version 8.2-1), metafor (version 4.8-0), tidyverse (version 2.0.0), and ggplot2. All other calculations will be performed in duplicate using Excel spreadsheets (Microsoft Excel for MAC, version 16.88).

## 3. Discussion

This study aims to provide the first comprehensive dose–response meta-analysis examining the relationship between baseline UI severity and male sling outcome. Findings will address a gap in the current literature by quantifying treatment effects across the continuum of UI severity rather than relying on arbitrary categorical comparisons.

The findings of this study have direct implications for clinical practice. First, by quantifying the relationship between baseline UI severity and sling outcomes across a continuous scale, this analysis may improve treatment decision-making when choosing between sling procedures and alternative treatments such as AUS. Importantly, identifying severity thresholds may help define clinically meaningful cutoffs beyond which sling efficacy declines substantially, thus guiding patient selection and improving preoperative counseling accuracy.

Currently, clinicians often rely on broad categorical recommendations (e.g., “slings are effective for mild-to-moderate incontinence”) without quantitative guidance on where moderate ends and severe begins [25]. Rather than relying on broad categories, clinicians may be able to better estimate the probability of treatment success for individual patients based on their specific severity level.

Second, the analysis will inform shared decision-making conversations between patients and surgeons. By providing quantitative estimates of success probability based on individual baseline UI severity, clinicians can facilitate more informed patient preferences. For example, a patient with borderline moderate-to-severe UI might learn that their predicted success rate is 45% with a sling versus 75% with an AUS, allowing them to weigh the trade-offs between less invasive surgery with lower success rates and more invasive surgery with higher efficacy. This approach aligns with the importance of aligning treatment selection with individual patient characteristics [24].

Third, detection of nonlinear relationships may reveal whether treatment effects decline gradually or demonstrate threshold effects. If a threshold exists, it might suggest a pathophysiologic boundary separating patients likely to benefit from urethral repositioning alone versus those requiring active compression (as provided by AUS). This could generate hypotheses for future mechanistic research and inform the development of treatment algorithms. The FORESEE meta-analysis [20] already demonstrated that baseline UI severity predicts outcomes but without exploring nonlinear association. A dose–response curve will enable more individualized risk stratification and address this limitation.

The use of advanced dose–response meta-analytic methods strengthen the reliability of the findings by efficiently integrating data across studies and accounting for heterogeneity. Flexible modeling approaches will be used to capture potential nonlinear relationships.

Comprehensive sensitivity analyses will evaluate the robustness of findings to methodological decisions. This transparency will allow readers to assess the stability of conclusions and identify potential limitations. Furthermore, the planned subgroup and meta-regression analyses will explore important clinical and methodological sources of heterogeneity. Comparison of Advance versus non-Advance slings is particularly relevant given that most evidence comes from Advance studies, and generalizability to other devices remains uncertain. Similarly, the assessment of whether study quality and publication bias influence findings will evaluate potential systematic bias in the evidence base.

### 3.1. Expected Outputs

Upon completion, this study will generate several outputs:

- A comprehensive systematic review and meta-analysis manuscript for submission to a high-impact urology or evidence synthesis medical journal according to PRISMA statement [30].
- Dose–response curves according to UI severity measures, with CIs and threshold estimates (if identified), describing the relationship between UI and sling failure probability.
- Possible dose–response moderators and specific estimates for pre-planned subgroups, if sufficient studies available.
- Evidence-based guidance for patient selection, facilitating individualized risk stratification, and supporting shared decision-making conversations between clinicians and patients considering surgical treatment for male UI.
- Quantitative evidence for guideline developers to inform treatment decisions for post-prostatectomy UI.
- Recommendations for future primary research, including optimal UI severity categorizations and essential covariates to measure.

### 3.2. Limitations

Some methodological limitations in the conduct of this review can be anticipated. As this review is based on observational studies rather than experimental designs, it is potentially subject to biases due to exposure misclassification or unmeasured confounding. The first issue may affect particularly studies that used less reliable methods to assess UI severity, that is, pad count, but also studies using very wide categories, reporting only ranges, and with category medians unavailable. These issues can distort the shape of the curve, especially for nonlinear models (e.g., restricted cubic splines). The second issue may particularly affect studies that did not adjust for pelvic irradiation, although, covariate adjustment did not appear to influence the overall baseline UI effect in a previous meta-analysis [20].

The clinical meaningfulness of summary results may be limited by heterogeneity across studies, particularly in sling type, UI etiology, and measurement of exposure and outcomes. We decided to use the longest available follow-up as the primary endpoint because it reflects more stable outcomes; however, we acknowledge that this choice may introduce heterogeneity. To address this limitation, we have incorporated some methodological safeguards: follow-up duration will be explored as a source of heterogeneity through meta-regression and sensitivity analyses will include comparisons across predefined follow-up intervals (e.g., <12 vs. ≥12 months) where feasible.

We anticipate heterogeneity in outcome definitions across studies. However, its impact on the dose–response meta-analysis is expected to be limited for several reasons. First, dose–response analyses are primarily based on within-study relative comparisons across exposure levels, rather than absolute outcome definitions. Therefore, differences in outcome definitions across studies are less likely to bias the estimation of the dose–response relationship. Second, any misclassification arising from heterogeneous definitions is likely to be non-differential across severity categories within studies, which would tend to attenuate effect estimates rather than distort the shape of the dose–response relationship. Third, despite variability in operational definitions, the underlying construct (continence or clinically meaningful improvement) is expected to be broadly consistent across studies. Importantly, to further mitigate this limitation, we will apply a predefined hierarchical approach to outcome definitions and a harmonization strategy by recalculating outcomes when feasible. In addition, we will perform sensitivity analyses restricted to the most homogeneous outcome definitions (e.g., objective measures only) and will also explore outcome definition as a potential source of heterogeneity through subgroup analyses.

Missing or imprecise data are frequent in such reviews, although we expect this issue to be solved by requesting authors to offer relevant data.

This review will summarize results from randomized trials, prospective and retrospective cohort studies, and case series. However, these study designs differ substantially, including the different statistical estimate and different biases.

While 12 months is widely considered the standard for evaluating stable sling outcomes, we selected a minimum follow-up of 6 months because this time point is an accepted intermediate endpoint in male UI management and allows inclusion of a broader evidence base, while follow-up duration will be explored as a moderator in meta-regression and subgroup analysis when feasible.

We will synthesize separately unadjusted and adjusted effect sizes. Based on the results of a previous meta-analysis [20], while the quality assessment will require mandatory adjustment for prior pelvic irradiation, other clinically relevant factors such as prior urethral interventions or UI surgery will not be required due to the currently limited and moderate-certainty evidence supporting their independent prognostic role. Furthermore, as above-mentioned, FORESEE meta-analyses [20] reported minimal differences between pooled estimates derived from adjusted and unadjusted effect sizes, suggesting that the overall exposure–response relationship is unlikely to be substantially influenced by covariate adjustment. However, the presence or absence of additional important covariates will be considered in the data extraction, meta-regression analysis, and overall interpretation of findings.

Publication bias and small-study effects represent an additional potential limitation. In this regard, we planned a very broad literature search. Furthermore, we will assess these biases using graphical and statistical methods specifically adapted to dose–response meta-analysis, although such approaches have limited power, particularly when the number of studies is small. Therefore, the presence of publication bias cannot be definitively excluded and may influence the magnitude of the observed associations.

Finally, this analysis is based on aggregated (study-level) data rather than individual participant data. As a result, the ability to adjust for confounding variables is limited to those reported and adjusted for within the original studies. Differences in covariate adjustment across studies may introduce heterogeneity and limit the interpretation of adjusted estimates. The non-random allocation of sling procedures across different severity levels may introduce confounding by indication. The planned value of sophisticated nonlinear modeling may be limited if the available studies mostly report only two severity categories, wide open-ended strata, or poorly standardized severity measurements. Furthermore, the use of aggregated data precludes more refined modeling of individual-level dose–response relationships. Despite these limitations, we believe they are unlikely to invalidate the overall dose–response patterns observed.

## 4. Conclusions

Building upon the foundation established by the FORESEE meta-analysis on prognostic factors [20], this analysis will provide the next level of precision in evidence-based treatment selection. This study aims to provide quantitative and clinically actionable evidence on the relationship between baseline UI severity and sling outcomes, with direct implications for clinical practice. By identifying how the probability of treatment success changes across the continuum of UI severity, the results may support more accurate patient selection and improve preoperative counseling, thereby facilitating shared decision-making. The identification of potential severity thresholds beyond which sling efficacy declines may help guide treatment choice, favoring alternative interventions such as AUS in patients with more severe UI, eventually improving patient outcomes. This approach moves beyond categorical classification toward more individualized, evidence-based risk stratification, aligning with the principles of personalized medicine. Although this type of review has methodological limitations, we believe they will not be serious enough to affect its value. Ultimately, this work aims to advance evidence-based, patient-centered care for male UI serving as a future guide for both clinicians and patients.

## Figures and Tables

**Table 1:** PICOTS criteria for dose–response meta-analysis of baseline urinary incontinence severity and male sling efficacy.

Population (P)	Adult male patients (≥18 years) with stress or mixed urinary incontinence undergoing synthetic non-adjustable sling implantation: Any incontinence etiology allowed Any baseline incontinence severity level Minimum study sample size: 30 patients
Index prognostic factor (I)	Baseline urinary incontinence severity measured using objective metrics: Measured before sling implantation Analyzed across multiple (at least two) severity categories
Comparator (C)	Higher levels of baseline incontinence severity within male sling-implanted patients compared to the reference category (lowest incontinence severity group in each study).
Outcomes (O)	Treatment failure: Failure to achieve cure Failure to achieve the success (cure plus improvement)
Timing (T)	Preoperative assessment of UI severity to predict the outcome over a post-sling implantation follow-up of at least six months.
Setting (S)	Any healthcare setting where male sling surgery is performed: Tertiary referral centers University hospitals Community hospitals Specialized urology/incontinence centers Geographic settings: international (no geographic restrictions) Study designs: Randomized controlled trials Prospective cohort studies Retrospective cohort studies Case series

## Data Availability

The data presented in this study are available in the article and Supplementary Materials.

## Supplementary Materials

The following supporting information can be downloaded at: https://www.mdpi.com/article/10.3390/jcm15114140/s1, Table S1: PRISMA-P (Preferred Reporting Items for Systematic review and Meta-Analysis Protocols) 2015 checklist: recommended items to address in a systematic review protocol*; Table S2: Search Strategy for Systematic Review and Dose-Response Meta-Analysis—MEDLINE (via PubMed); Table S3: Search Strategy for Systematic Review and Dose-Response Meta-Analysis—Scopus; Table S4: Search Strategy for Systematic Review and Dose-Response Meta-Analysis—Web of Science (Core Collection); Table S5: Search Strategy for Systematic Review and Dose-Response Meta-Analysis—Cochrane Central Register of Controlled Trials (CENTRAL); Table S6: Risk of Bias categorization criteria for overall quality judgement of included papers based on QUIPS rating.

## Abbreviations

The following abbreviations are used in this manuscript:

AUS	artificial urinary sphincters
UI	urinary incontinence
VPC	Variance Partition Coefficient
