# Supplementary material for: Male Urinary Incontinence Severity and Non-Adjustable Sling Outcomes: Protocol for a Multivariate Dose–Response Meta-Analysis
Source: J Clin Med. 2026 May 27;15(11):4140. doi: 10.3390/jcm15114140 (PMC13257566; doi:10.3390/jcm15114140)
Supplement: Supplementary file 1 [file jcm-15-04140-s001.zip › Suppl Table S2-S5 - Search Strategy.pdf]

Supplementary Table S2. Search Strategy for Systematic Review and Dose-Response Meta-Analysis - MEDLINE (via PubMed)

#	Search Component	Search Terms
#1	Suburethral slings (MeSH)	"Suburethral Slings"[Mesh]
#2	Urologic surgical procedures (MeSH)	"Urologic Surgical Procedures, Male"[Mesh]
#3	Surgical mesh (MeSH)	"Surgical Mesh"[Mesh]
#4	Combine MeSH intervention terms	#1 OR (#2 AND #3)
#5	Male sling (free-text)	"male sling*"[tiab] OR "masculin* sling*"[tiab] OR "men sling*"[tiab] OR "men's sling*"[tiab]
#6	Suburethral/retrourethral sling (free-text)	"retrourethral sling*"[tiab] OR "retro-urethral sling*"[tiab] OR "perineal sling*"[tiab] OR "bulbar sling*"[tiab] OR "bulbourethral sling*"[tiab]
#7	Transobturator sling (free-text)	"transobturator sling*"[tiab] OR "trans-obturator sling*"[tiab] OR "transobturator male sling*"[tiab] OR TOMS[tiab] OR "readjustment sling*"[tiab] OR "repositioning sling*"[tiab]
#8	Bone-anchored sling (free-text)	"bone anchor* sling*"[tiab] OR "bone-anchor* sling*"[tiab] OR BAMS[tiab] OR "bone fixed sling*"[tiab] OR "bone fixation sling*"[tiab]
#9	Specific device names	Advance[tiab] OR AdVance[tiab] OR "AdVance XP"[tiab] OR "Advance XP"[tiab] OR InVance[tiab] OR "In-Vance"[tiab] OR Virtue[tiab] OR "I-Stop"[tiab] OR "I Stop"[tiab] OR "IStop"[tiab] OR Tiloop[tiab] OR "Ti-loop"[tiab] OR "Ti loop"[tiab] OR Surgymesh[tiab] OR "Surgy mesh"[tiab] OR "Argus sling"[tiab] OR "male Remeex"[tiab] OR "Remeex system"[tiab] OR "ATOMS"[tiab] OR M-sling[tiab] OR M-Sling[tiab]
#10	Retroluminal/compression terms	"retroluminal repositioning"[tiab] OR "urethral support*"[tiab] OR "urethral compression"[tiab] OR "urethral suspension"[tiab]
#11	Combine all sling intervention terms	#4 OR #5 OR #6 OR #7 OR #8 OR #9 OR #10
#12	Male population filter	(male*[tiab] OR men[tiab] OR man[tiab] OR "Male"[Mesh])
#13	Incontinence context	(incontinen*[tiab] OR "Urinary Incontinence"[Mesh] OR "urinary leakage"[tiab] OR "stress incontinence"[tiab])
#14	Combine sling AND male AND incontinence	#11 AND #12 AND #13
#15	Post-prostatectomy specific	("post prostatectomy"[tiab] OR "postprostatectomy"[tiab] OR "post-prostatectomy"[tiab] OR "after prostatectomy"[tiab] OR

#	Search Component	Search Terms
		"following prostatectomy"[tiab] OR "radical prostatectomy"[tiab] OR "Prostatectomy"[Mesh])
#16	Sling with post-prostatectomy context	#11 AND #15
#17	Combine male incontinence sling OR post-prostatectomy sling	#14 OR #16
#18	Exclude: Female/vaginal slings	"vaginal sling*"[tiab] OR "female sling*"[tiab] OR "women sling*"[tiab] OR "women's sling*"[tiab] OR "midurethral sling*"[tiab] OR "mid-urethral sling*"[tiab] OR "tension-free vaginal tape"[tiab] OR "TVT"[tiab] OR "TOT"[tiab] OR "transobturator tape"[tiab] OR "retropubic sling"[tiab] OR "Burch"[tiab] OR "female stress"[tiab]
#19	Exclude: Publication types (MeSH)	"Letter"[Publication Type] OR "Editorial"[Publication Type] OR "Comment"[Publication Type]
#20	Exclude: Publication types (free-text)	correction[ti] OR addendum[ti] OR "letter to"[ti] OR "reply to"[ti] OR "author response"[ti]
#21	Combine all exclusions	#18 OR #19 OR #20
#22	Final search	#17 NOT #21

Final PubMed Search String

((("Suburethral Slings"[Mesh]) OR ("Urologic Surgical Procedures, Male"[Mesh] AND "Surgical Mesh"[Mesh]) OR ("male sling*"[tiab] OR "masculin* sling*"[tiab] OR "men sling*"[tiab] OR "men's sling*"[tiab]) OR ("retrourethral sling*"[tiab] OR "retro-urethral sling*"[tiab] OR "perineal sling*"[tiab] OR "bulbar sling*"[tiab] OR "bulbourethral sling*"[tiab]) OR ("transobturator sling*"[tiab] OR "trans-obturator sling*"[tiab] OR "transobturator male sling*"[tiab] OR TOMS[tiab] OR "readjustment sling*"[tiab] OR "repositioning sling*"[tiab]) OR ("bone anchor* sling*"[tiab] OR "bone-anchor* sling*"[tiab] OR BAMS[tiab] OR "bone fixed sling*"[tiab] OR "bone fixation sling*"[tiab]) OR (Advance[tiab] OR AdVance[tiab] OR "AdVance XP"[tiab] OR "Advance XP"[tiab] OR InVance[tiab] OR "In-Vance"[tiab] OR Virtue[tiab] OR "I-Stop"[tiab] OR "I Stop"[tiab] OR "IStop"[tiab] OR Tiloop[tiab] OR "Ti-loop"[tiab] OR "Ti loop"[tiab] OR Surgymesh[tiab] OR "Surgy mesh"[tiab] OR "Argus sling"[tiab] OR "male Remeex"[tiab] OR "Remeex system"[tiab]) OR ("retroluminal repositioning"[tiab] OR "urethral support*"[tiab] OR "urethral compression"[tiab] OR "urethral suspension"[tiab])) AND ((male*[tiab] OR men[tiab] OR man[tiab] OR "Male"[Mesh]) AND (incontinen*[tiab] OR "Urinary Incontinence"[Mesh] OR "urinary leakage"[tiab] OR "stress incontinence"[tiab])) OR ((("Suburethral Slings"[Mesh]) OR ("Urologic Surgical Procedures, Male"[Mesh] AND "Surgical Mesh"[Mesh]) OR ("male sling*"[tiab] OR "masculin* sling*"[tiab] OR "men sling*"[tiab] OR "men's sling*"[tiab]) OR ("suburethral sling*"[tiab] OR "sub-urethral sling*"[tiab] OR "retrourethral sling*"[tiab] OR "retro-urethral sling*"[tiab] OR "perineal sling*"[tiab] OR "bulbar sling*"[tiab] OR "bulbourethral sling*"[tiab]) OR ("transobturator sling*"[tiab] OR "trans-obturator sling*"[tiab] OR "transobturator male sling*"[tiab] OR TOMS[tiab] OR "readjustment sling*"[tiab] OR "repositioning sling*"[tiab]) OR ("bone anchor* sling*"[tiab] OR "bone-anchor* sling*"[tiab] OR BAMS[tiab] OR "bone fixed sling*"[tiab] OR "bone fixation sling*"[tiab]) OR (Advance[tiab] OR AdVance[tiab] OR "AdVance XP"[tiab] OR "Advance

XP"[tiab] OR InVance[tiab] OR "In-Vance"[tiab] OR Virtue[tiab] OR "I-Stop"[tiab] OR "I Stop"[tiab] OR "IStop"[tiab] OR Tiloop[tiab] OR "Ti-loop"[tiab] OR "Ti loop"[tiab] OR Surgymesh[tiab] OR "Surgy mesh"[tiab] OR "Argus sling"[tiab] OR "male Remeex"[tiab] OR "Remeex system"[tiab]) OR "ATOMS"[tiab] OR M-sling[tiab] OR M-Sling[tiab] OR ("retroluminal repositioning"[tiab] OR "urethral support*"[tiab] OR "urethral compression"[tiab] OR "urethral suspension"[tiab])) AND ("post prostatectomy"[tiab] OR "postprostatectomy"[tiab] OR "post-prostatectomy"[tiab] OR "after prostatectomy"[tiab] OR "following prostatectomy"[tiab] OR "radical prostatectomy"[tiab] OR "Prostatectomy"[Mesh]))) NOT ("vaginal sling*"[tiab] OR "female sling*"[tiab] OR "women sling*"[tiab] OR "women's sling*"[tiab] OR "midurethral sling*"[tiab] OR "mid-urethral sling*"[tiab] OR "tension-free vaginal tape"[tiab] OR "TVT"[tiab] OR "TOT"[tiab] OR "transobturator tape"[tiab] OR "retropubic sling"[tiab] OR "Burch"[tiab] OR "female stress"[tiab] OR "Letter"[Publication Type] OR "Editorial"[Publication Type] OR "Comment"[Publication Type] OR correction[ti] OR addendum[ti] OR "letter to"[ti] OR "reply to"[ti] OR "author response"[ti])

Supplementary Table S3. Search Strategy for Systematic Review and Dose-Response Meta-Analysis - Scopus

#	Search Component	Search Terms
#1	Male sling terms	TITLE-ABS-KEY("male sling*" OR "masculin* sling*" OR "men sling*" OR "men's sling*")
#2	Suburethral/retrourethral sling	TITLE-ABS-KEY("suburethral sling*" OR "sub-urethral sling*" OR "retrourethral sling*" OR "retro-urethral sling*" OR "perineal sling*" OR "bulbar sling*" OR "bulbourethral sling*")
#3	Transobturator sling	TITLE-ABS-KEY("transobturator sling*" OR "trans-otturatore sling*" OR "transobturator male sling*" OR TOMS OR "readjustment sling*" OR "repositioning sling*")
#4	Bone-anchored sling	TITLE-ABS-KEY("bone anchor* sling*" OR "bone-anchor* sling*" OR BAMS OR "bone fixed sling*" OR "bone fixation sling*")
#5	Specific device names	TITLE-ABS-KEY("Advance sling" OR "AdVance sling" OR "AdVance XP" OR "Advance XP" OR "AdvanceXP" OR "AdVanceXP" OR InVance OR "In-Vance" OR Virtue OR "I-Stop" OR "I Stop" OR IStop OR Tiloop OR "Ti-loop" OR "Ti loop" OR Surgymesh OR "Surgy mesh" OR "Argus sling" OR "male Remeex" OR "Remeex system" OR "ATOMS" OR "M-sling" OR "M-Sling")
#6	Retroluminal/compression	TITLE-ABS-KEY("retroluminal repositioning" OR "urethral compression" OR "urethral suspension")
#7	Sling procedure with male context	TITLE-ABS-KEY("sling procedure*" OR "sling implant*" OR "sling surgery" OR "sling operation*") AND TITLE-ABS-KEY(male* OR men OR "post-prostatectomy" OR postprostatectomy) AND TITLE-ABS-KEY(incontinen* OR "urinary leakage")
#8	Combine all sling terms	#1 OR #2 OR #3 OR #4 OR #5 OR #6 OR #7

#	Search Component	Search Terms
#9	Incontinence treatment context	TITLE-ABS-KEY(incontinen* OR "urinary leakage" OR "urine leakage" OR "stress urinary" OR "continence surgery" OR "anti-incontinence" OR "incontinence treatment" OR "incontinence management")
#10	Post-prostatectomy context	TITLE-ABS-KEY("post prostatectomy" OR postprostatectomy OR "post-prostatectomy" OR "after prostatectomy" OR "following prostatectomy" OR "radical prostatectomy" OR prostatectomy)
#12	Sling AND (male incontinence OR post-prostatectomy)	#8 AND (#9 OR #10)
#13	Exclude: Female/vaginal slings	TITLE-ABS-KEY("vaginal sling*" OR "female sling*" OR "women sling*" OR "women's sling*" OR "midurethral sling*" OR "mid-urethral sling*" OR "mid urethral sling*" OR "tension-free vaginal tape" OR "TVT" OR "TOT" OR "retropubic sling" OR "Burch" OR "female stress incontinence" OR "female urinary incontinence" OR "women with" OR "in women" OR "female" OR "postpartum" OR "post-partum" OR "obstetric")
#14	Exclude: Publication types	DOCTYPE(le OR ed OR no OR bk OR er OR cp) OR TITLE(correction OR addendum OR erratum OR "letter to" OR "reply to" OR "author response" OR commentary OR protocol OR "study protocol")
#15	Combine exclusions	#13 OR #14
#16	Final search	#12 AND NOT #15

Final Scopus Search String

(TITLE-ABS-KEY ("male sling*" OR "masculin* sling*" OR "men sling*" OR "men's sling*") OR TITLE-ABS-KEY ("suburethral sling*" OR "sub-urethral sling*" OR "retrourethral sling*" OR "retro-urethral sling*" OR "perineal sling*" OR "bulbar sling*" OR "bulbourethral sling*") OR TITLE-ABS-KEY ("transobturator sling*" OR "trans-otturatore sling*" OR "transobturator male sling*" OR TOMS OR "readjustment sling*" OR "repositioning sling*") OR TITLE-ABS-KEY ("bone anchor* sling*" OR "bone-anchor* sling*" OR BAMS OR "bone fixed sling*" OR "bone fixation sling*") OR TITLE-ABS-KEY ("Advance sling" OR "AdVance sling" OR "AdVance XP" OR "Advance XP" OR "AdvanceXP" OR "AdVanceXP" OR InVance OR "In-Vance" OR "Virtue sling" OR "I-Stop" OR "I Stop" OR IStop OR Tiloop OR "Ti-loop" OR "Ti loop" OR Surgymesh OR "Surgy mesh" OR "Argus sling" OR "male Remeex" OR "Remeex system" OR "ATOMS" OR "M-sling" OR "M-Sling") OR TITLE-ABS-KEY ("retroluminal repositioning" OR "urethral compression" OR "urethral suspension") OR (TITLE-ABS-KEY ("sling procedure*" OR "sling implant*" OR "sling surgery" OR "sling operation*") AND TITLE-ABS-KEY (male* OR men OR "post-prostatectomy" OR postprostatectomy) AND TITLE-ABS-KEY (incontinen* OR "urinary leakage"))) AND (TITLE-ABS-KEY (incontinen* OR "urinary leakage" OR "urine leakage" OR "stress urinary" OR "continence surgery" OR "anti-incontinence" OR "incontinence treatment" OR "incontinence management") OR TITLE-ABS-KEY ("post prostatectomy" OR postprostatectomy OR "post-prostatectomy" OR "after prostatectomy" OR "following prostatectomy" OR "radical prostatectomy" OR prostatectomy)) AND NOT (TITLE-ABS-KEY ("vaginal sling*" OR "female sling*" OR "women sling*" OR "women's

sling*" OR "midurethral sling*" OR "mid-urethral sling*" OR "mid urethral sling*" OR "tension-free vaginal tape" OR "TVT" OR "TOT" OR "retropubic sling" OR "Burch" OR "female stress incontinence" OR "female urinary incontinence" OR "women with" OR "in women" OR "female" OR "postpartum" OR "post-partum" OR "obstetric") OR DOCTYPE (le OR ed OR no OR bk OR er OR cp) OR TITLE (correction OR addendum OR erratum OR "letter to" OR "reply to" OR "author response" OR commentary OR protocol OR "study protocol"))
Filters: None (all years, all languages)

Supplementary Table S4. Search Strategy for Systematic Review and Dose-Response Meta-Analysis - Web of Science (Core Collection)

#	Search Component	Search Terms
#1	Male sling terms	TS=("male sling*" OR "men sling*" OR "men's sling*" OR "masculine sling*")
#2	Suburethral/retrourethral sling	TS=("suburethral sling*" OR "sub-urethral sling*" OR "retrourethral sling*" OR "retro-urethral sling*" OR "perineal sling*" OR "bulbar sling*" OR "bulbourethral sling*")
#3	Transobturator sling	TS=("transobturator sling*" OR "trans-obturator sling*" OR "transobturator male sling*" OR "TOMS" OR "readjustment sling*" OR "repositioning sling*")
#4	Bone-anchored sling	TS=("bone anchor* sling*" OR "bone-anchor* sling*" OR "bone fixed sling*" OR "bone fixation sling*")
#5	Specific device names - Group 1	TS=(AdVance OR "AdVance XP" OR "Advance XP" OR InVance OR "In-Vance" OR Virtue)
#6	Specific device names - Group 2	TS=("I-Stop" OR "I Stop" OR IStop OR Tiloop OR "Ti-loop" OR "Ti loop" OR Surgymesh OR "Surgy mesh")
#7	Specific device names - Group 3	TS=("Argus sling" OR "male Remeex" OR "Remeex system" OR “ATOMS” OR “M-Sling” OR “M-Sling”)
#8	Combine all sling terms	#1 OR #2 OR #3 OR #4 OR #5 OR #6 OR #7
#9	Incontinence terms	TS=((male* OR men OR man) NEAR/5 (incontinen* OR "urinary leakage" OR "stress incontinence"))
#10	Sling AND (male incontinence)	#8 AND #9
#11	Exclude: Female/vaginal slings - Group 1	TS=("vaginal sling*" OR "female sling*" OR "women sling*" OR "women's sling*")
#12	Exclude: Female/vaginal slings - Group 2	TS=("midurethral sling*" OR "mid-urethral sling*" OR "tension-free vaginal tape" OR "retropubic sling" OR Burch OR "female stress")
#13	Exclude: Publication types	DT=(Correction, Addition OR Editorial Material OR Letter OR Note)
#14	Exclude: Title terms	TI=(correction OR addendum OR "letter to" OR "reply to" OR "author response")
#15	Combine all exclusions	#11 OR #12 OR #13 OR #14

#	Search Component	Search Terms
#16	Final search	#10 NOT #15

((((((((TS=("male sling*" OR "masculin* sling*" OR "men sling*" OR "men's sling*") OR TS=("suburethral sling*" OR "sub-urethral sling*" OR "retrourethral sling*" OR "retro-urethral sling*" OR "perineal sling*" OR "bulbar sling*" OR "bulbourethral sling*") OR TS=("transobturator sling*" OR "trans-obturator sling*" OR "transobturator male sling*" OR "TOMS" OR "readjustment sling*" OR "repositioning sling*") OR TS=("bone anchor* sling*" OR "bone-anchor* sling*" OR "BAMS" OR "bone fixed sling*" OR "bone fixation sling*") OR TS=(Advance OR AdVance OR "AdVance XP" OR "Advance XP" OR InVance OR "In-Vance" OR Virtue OR "I-Stop" OR "I Stop" OR IStop OR Tiloop OR "Ti-loop" OR "Ti loop" OR Surgymesh OR "Surgy mesh" OR "Argus sling" OR "male Remeex" OR "Remeex system" OR "ATOMS" OR “M-sling” OR M-Sling))) AND (TS=((male* OR men OR man) NEAR/5 (incontinen* OR "urinary leakage" OR "stress incontinence")))) NOT (TS=("vaginal sling*" OR "female sling*" OR "women sling*" OR "women's sling*" OR "midurethral sling*" OR "mid-urethral sling*" OR "tension-free vaginal tape" OR "TVT" OR "retropubic sling" OR "Burch" OR "female stress") OR DT=(Letter OR Editorial OR Correction OR "Book Review") OR TI=(correction OR addendum OR "letter to" OR "reply to" OR "author response")))))))
Filters: None (all years, all languages)

Supplementary Table S5. Search Strategy for Systematic Review and Dose-Response Meta-Analysis - Cochrane Central Register of Controlled Trials (CENTRAL)

#	Search Component	Search Terms
#1	Suburethral slings (MeSH)	[mh "Suburethral Slings"]
#2	Urologic procedures (MeSH)	[mh "Urologic Surgical Procedures, Male"]
#3	Surgical mesh (MeSH)	[mh "Surgical Mesh"]
#4	Combine MeSH terms	#1 OR (#2 AND #3)
#5	Male sling (free-text)	("male sling" OR "masculin sling" OR "men sling" OR "men's sling"):ti,ab,kw
#6	Suburethral/retrourethral sling	("suburethral sling" OR "sub-urethral sling" OR "retrourethral sling" OR "retro-urethral sling" OR "perineal sling" OR "bulbar sling" OR "bulbourethral sling"):ti,ab,kw
#7	Transobturator sling	("transobturator sling" OR "trans-obturator sling" OR "transobturator male sling" OR TOMS OR "readjustment sling" OR "repositioning sling"):ti,ab,kw
#8	Bone-anchored sling	("bone anchor sling" OR "bone-anchor sling" OR BAMS OR "bone fixed sling" OR "bone fixation sling"):ti,ab,kw
#9	Specific device names	("Advance sling" OR "AdVance sling" OR "AdVance XP" OR "Advance XP" OR "AdVanceXP" OR "AdvanceXP" OR InVance OR "In-Vance" OR Virtue OR "I-Stop" OR "I Stop" OR IStop OR Tiloop OR "Ti-loop" OR "Ti loop" OR Surgymesh OR "Surgy mesh" OR "Argus sling" OR "male Remeex" OR "Remeex system"):ti,ab,kw

#	Search Component	Search Terms
#10	Retroluminal/compression	("retroluminal repositioning" OR "urethral compression" OR "urethral suspension"):ti,ab,kw
#11	Combine all sling intervention terms	#4 OR #5 OR #6 OR #7 OR #8 OR #9 OR #10
#12	Exclude: Female/vaginal slings	("vaginal sling" OR "female sling" OR "women sling" OR "women's sling" OR "midurethral sling" OR "mid-urethral sling" OR "tension-free vaginal tape" OR TVT OR TOT OR "retropubic sling" OR Burch OR "female stress" OR "female" OR "postpartum" OR "post-partum" OR "obstetric"):ti,ab,kw
#13	Exclude: Publication types	(correction OR addendum OR "letter to" OR "reply to" OR "author response"):ti
#14	Combine exclusions	#12 OR #13
#15	Final search	#11 NOT #14

Final CENTRAL Search String

(([mh "Suburethral Slings"] OR ([mh "Urologic Surgical Procedures, Male"] AND [mh "Surgical Mesh"]) OR ("male sling*" OR "masculin* sling*" OR "men sling*" OR "men's sling*"):ti,ab,kw OR ("suburethral sling*" OR "sub-urethral sling*" OR "retrourethral sling*" OR "retro-urethral sling*" OR "perineal sling*" OR "bulbar sling*" OR "bulbourethral sling*"):ti,ab,kw OR ("transobturator sling*" OR "trans-obturator sling*" OR "transobturator male sling*" OR TOMS OR "readjustment sling*" OR "repositioning sling*"):ti,ab,kw OR ("bone anchor* sling*" OR "bone-anchor* sling*" OR BAMS OR "bone fixed sling*" OR "bone fixation sling*"):ti,ab,kw OR (Advance OR AdVance OR "AdVance XP" OR "Advance XP" OR InVance OR "In-Vance" OR Virtue OR "I-Stop" OR "I Stop" OR IStop OR Tiloop OR "Ti-loop" OR "Ti loop" OR Surgymesh OR "Surgy mesh" OR "Argus sling" OR "male Remeex" OR "Remeex system"):ti,ab,kw OR ("retroluminal repositioning" OR "urethral support*" OR "urethral compression" OR "urethral suspension"):ti,ab,kw) NOT (("vaginal sling*" OR "female sling*" OR "women sling*" OR "women's sling*" OR "midurethral sling*" OR "mid-urethral sling*" OR "tension-free vaginal tape" OR TVT OR TOT OR "retropubic sling" OR Burch OR "female stress"):ti,ab,kw OR (correction OR addendum OR "letter to" OR "reply to" OR "author response"):ti)

Limits: Trials (CENTRAL default)
