# Supplementary material for: Male Urinary Incontinence Severity and Non-Adjustable Sling Outcomes: Protocol for a Multivariate Dose–Response Meta-Analysis
Source: J Clin Med. 2026 May 27;15(11):4140. doi: 10.3390/jcm15114140 (PMC13257566; doi:10.3390/jcm15114140)
Supplement: Supplementary file 1 [file jcm-15-04140-s001.zip › Suppl Table S6 - Risk of bias.pdf]

Supplementary Table S6. Risk of Bias categorization criteria for overall quality judgement of included papers based on QUIPS rating

Domains rating	Overall judgement
All “low” or up to one “moderate”	“low”
Two or more “moderate”	“moderate”
Any “high”	“high”
